# Welcome New Members of the *Neurophotonics* Editorial Board!

**DOI:** 10.1117/1.NPh.8.2.020101

**Published:** 2021-06-22

**Authors:** Anna Devor

## Abstract

Neurophotonics Editor in Chief Anna Devor welcomes an expanded editorial board and announces three Special Sections for the coming year.

I made my decision to image the brain when I started my training in neuroscience as an undergraduate student at Hebrew University of Jerusalem in Israel. How long ago was it? It was so long ago, that two-photon microscopy was not yet invented, genetically encoded fluorescent probes did not exist, and a 12x12 photodiode array was considered state-of-the-art instrumentation for space-resolved imaging of brain activity. Today, neurophotonic technologies are evolving faster than ever, driving a revolution in neuroscience. These advances are not only deepening our basic knowledge of how a healthy brain works, but also profoundly impacting our understanding of brain disease and guiding new diagnostic and therapeutic strategies. The same optical tools have utility more broadly in all biological sciences.

The mission of *Neurophotonics* is to facilitate the continuous development, evolution, and broadest possible application of impactful optical technologies in neuroscience in particular, but also in biology and medicine in general. At *Neurophotonics*, we aim to provide a natural home for your research whether you are a technology expert developing novel optical tools or a biology expert applying these tools to address impactful neuroscience questions.

We realize that each of these domains is intrinsically and massively diverse. Therefore, we have expanded our editorial board to reflect the breadth of the neurophotonic arena spanning across scales, imaging modalities, and species—from animals to humans.

**Figure:**
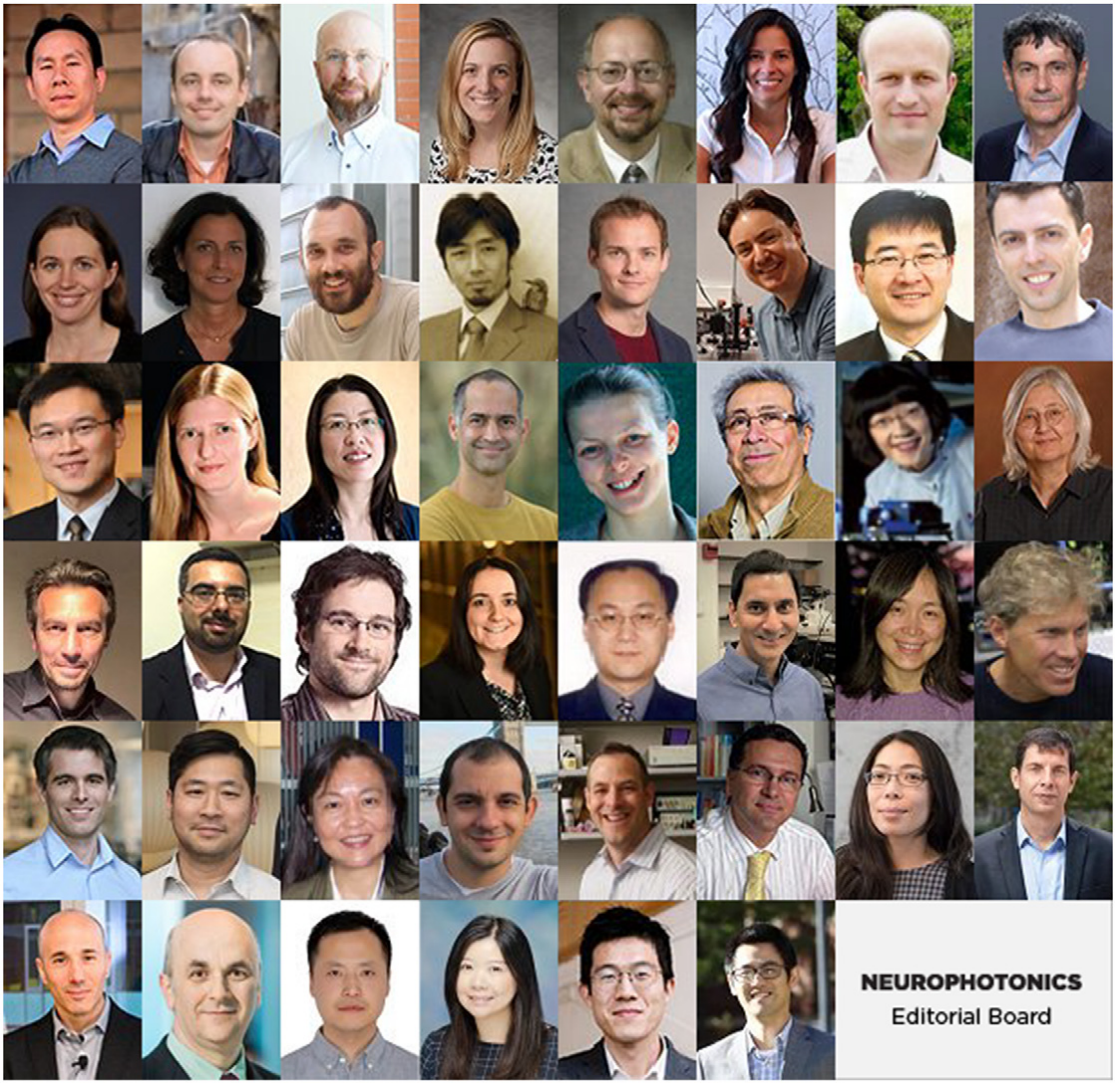
Please, meet our editorial board members and learn more about their expertise at https://www.spiedigitallibrary.org/journals/neurophotonics/editorial-board.

*I am delighted to extend our warm welcome to the newly appointed members with outstanding scientific qualifications and express my appreciation to those who continue providing invaluable service to the journal.*

While I have your attention, I would like to mention that we have three Special Sections open for submission: (1) Hybrid Photonic/X Neurointerfaces, (2) Computational Approaches for Neuroimaging, and (3) Imaging Neurovascular, Neuroglial, and Neuroimmune Interfaces.

**Figure:**
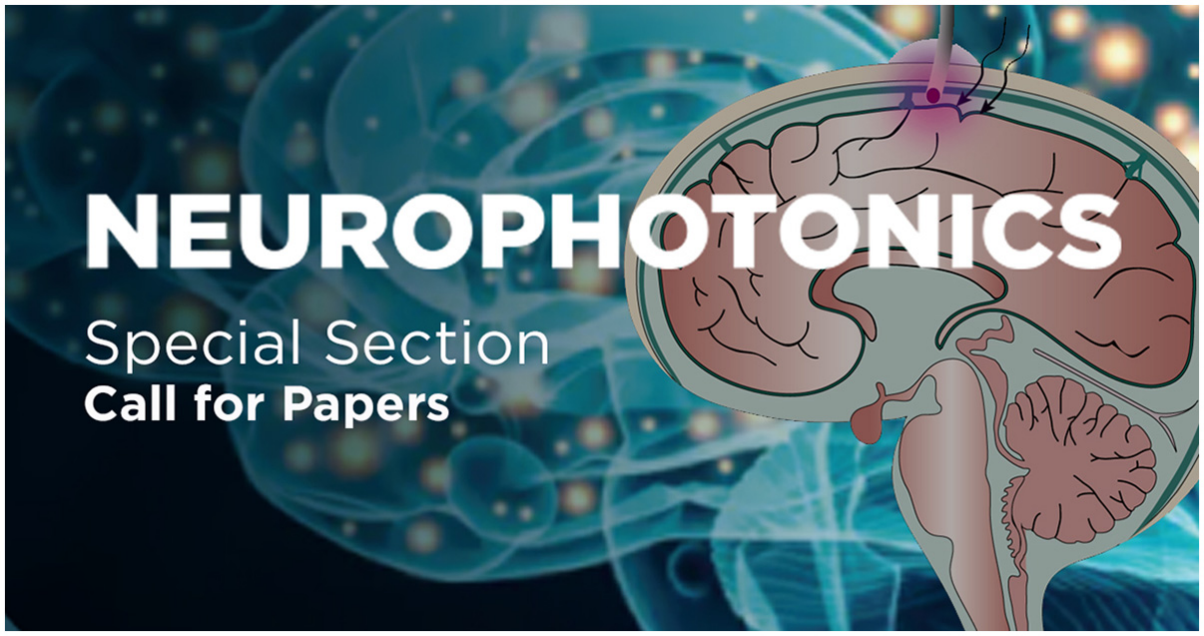
Do you combine optics and photonics with ultrasound, transparent electronics, MRI, or other technologies to study the brain? Submit to the **Special Section on Hybrid Photonic/X Neurointerfaces**!

**Figure:**
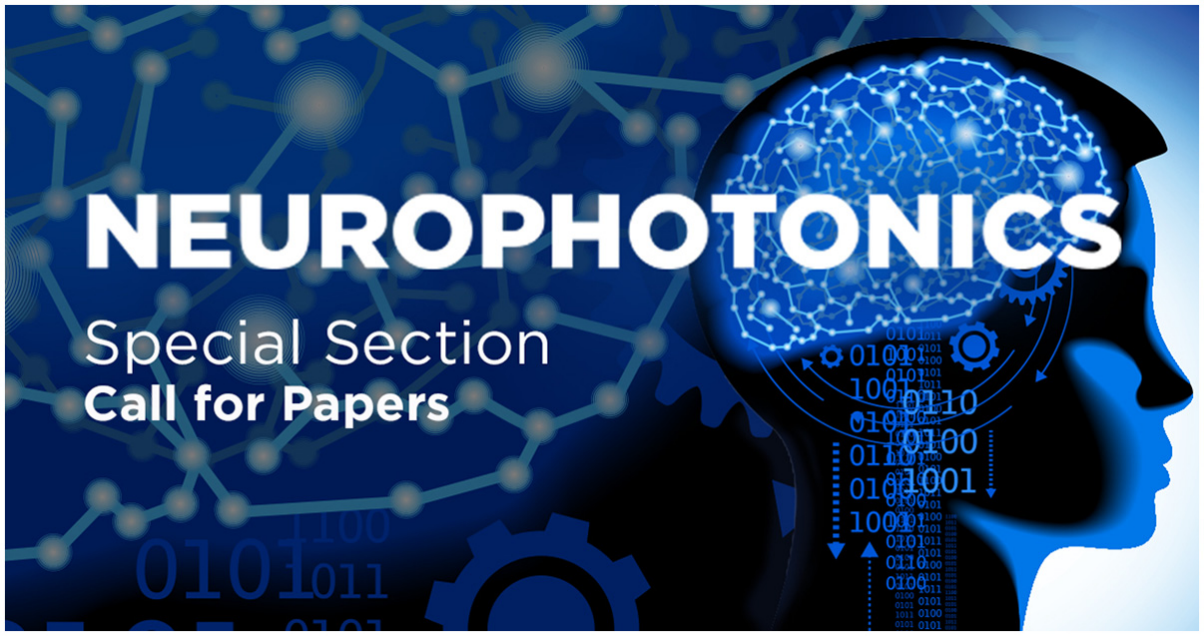
Do you develop and apply computational approaches to acquire, reconstruct, analyze, and interpret optical imaging data? Submit to the **Special Section on Computational Approaches for Neuroimaging**!

**Figure:**
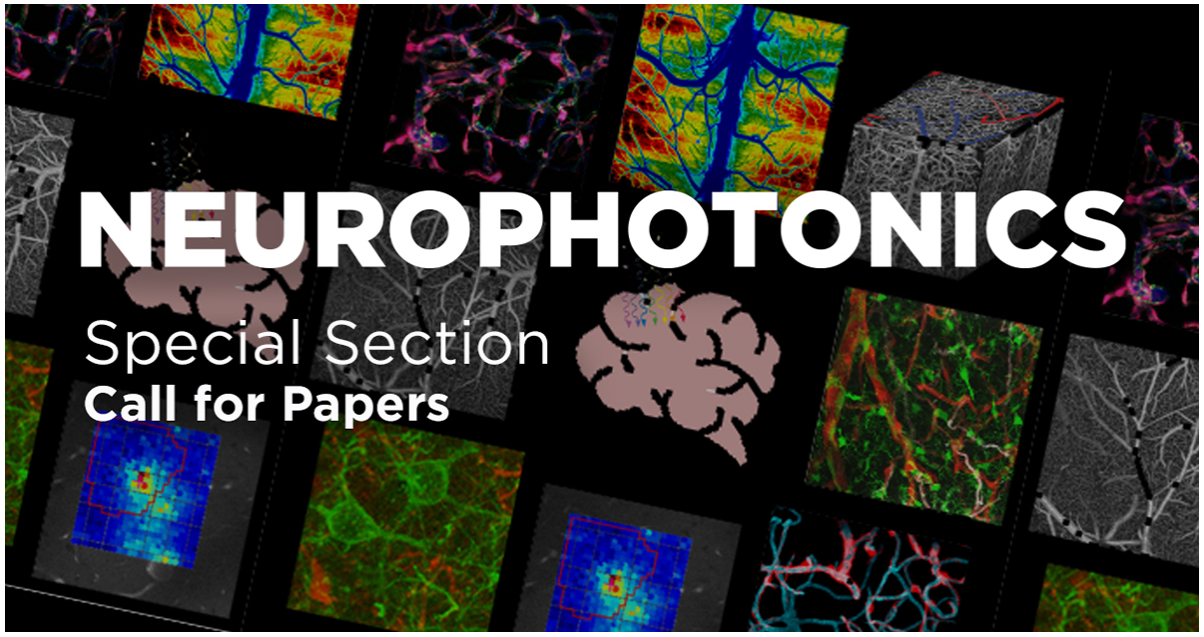
Do you use neurophotonics to study neurovascular, neuroglial, neurometabolic, and neuroimmune interactions? Submit to the **Special Section on Imaging Neurovascular, Neuroglial, and Neuroimmune Interfaces**!

As always, we love hearing your feedback. If you would like to propose another specific Special Section or contribute a topical Review or Primer, please let us know by emailing our editorial offices, neurophotonics@spie.org. We look forward to hearing from you and invite you to send your best work to *Neurophotonics*!

